# Circulating MicroRNA Profile as a Potential Predictive Biomarker for Early Diagnosis of Spontaneous Abortion in Patients With Subclinical Hypothyroidism

**DOI:** 10.3389/fendo.2018.00128

**Published:** 2018-04-06

**Authors:** Yingying Zhou, Xinyi Wang, Yuanyuan Zhang, Tong Zhao, Zhongyan Shan, Weiping Teng

**Affiliations:** ^1^Department of Endocrinology and Metabolism, Institute of Endocrinology, Liaoning Provincial Key Laboratory of Endocrine Diseases, The First Affiliated Hospital of China Medical University, Heping Distinct, Shenyang, Liaoning, China; ^2^Division of Endocrinology, Department of Internal Medicine, The First Affiliated Hospital of Zhengzhou University, Zhengzhou, China; ^3^Department of Laboratory Medicine, The First Affiliated Hospital of China Medical University, Shenyang, China; ^4^Beijing Key Laboratory of Diabetes Prevention and Research, Department of Endocrinology, Lu He Hospital, Capital Medical University, Beijing, China

**Keywords:** circulating microRNA, subclinical hypothyroidism, spontaneous abortion, biomarker, diagnosis

## Abstract

An increasing number of studies suggest that subclinical hypothyroidism (SCH) is associated with complications of gestation, including spontaneous abortion (SA). However, the underlying mechanism is not clear. MicroRNA (miRNA) has been demonstrated to be closely related to gynecological reproductive diseases. We determined miRNA expression in patients with SCH, SCH with SA (SCH + SA), and in those with SA as well as healthy controls (HCs), and analyzed whether dysregulation in several miRNAs was specific to these cohorts. An Agilent Human miRNA array was used to explore miRNA levels in pooled serum samples as a pilot study, followed by a validation of selected miRNAs by real-time polymerase chain reaction in SCH (*N* = 24), SA (*N* = 19), SCH + SA (*N* = 21), and HC cohorts (*N* = 18). The relative expression of miR-940 was elevated in the SCH + SA group compared with SCH, SA, and HC groups. In addition, miR-486-5p was upregulated in the SCH + SA group compared with SA and HC groups, without a difference noted between SCH + SA and SCH groups. Further analysis suggested that miR-940 or miR-486-5p may be potential predictive biomarkers for the early diagnosis of SA in patients with SCH.

## Introduction

Subclinical hypothyroidism (SCH) is the most common form of thyroid hormone deficiency (1). During pregnancy, SCH is characterized by elevated levels of serum thyroid-stimulating hormone (TSH) above the upper reference limit of the gestational maternal-specific reference (2) with normal free thyroid hormones. Some studies have linked SCH to gestational complications, including gestational hypertension (3), the prelabor rupture of membranes (3), preterm delivery (4), and spontaneous abortion (SA) (5). Although the newly published guidelines by the American Thyroid Association revised the upper limit of the TSH reference for the first trimester (6), epidemiological studies reported that women in early pregnancy with a serum TSH level between 2.5 mIU/L and the gestational-specific reference were more prone to SA (7, 8). However, since the causes of SA are multiple and complicated, studies failed to identify the mechanism(s) involved in the occurrence of SCH with SA from either disease.

MicroRNAs (miRNAs), a class of conservative, endogenous small non-coding RNAs, only 19–24 nucleotides in length, usually negatively regulate the expression of hundreds of genes by binding to the 3′-untranslated region (UTR) or 5′-UTR of their target mRNAs (9, 10). miRNAs play a fundamental role in controlling various cellular functions, such as metabolism, apoptosis, and differentiation. miRNAs have been stably detected in extracellular fluids (11, 12), including plasma/serum, while circulating miRNAs seem to be resistant to endogenous ribonuclease activity (12); they have emerged as biomarkers for the diagnosis of various diseases (13). Dysfunction of miRNA expression in the villous and decidua was an underlying factor in the pathogenesis of SA by influencing angiogenesis (14), proliferation, invasion or apoptosis (15–18). In addition, studies have also suggested that circulating miRNAs may be potential molecular biomarkers in the pathophysiological evolution of pregnancy, including SA (19, 20). However, studies investigating miRNA expression profiles in the pathogenesis of abortion associated with SCH are lacking.

The aim of our study was to characterize miRNA expression patterns in SCH patients, with or without SA, in SA patients and in healthy controls (HCs), which may help us find miRNAs specific to the four different groups.

## Materials and Methods

### Study Population and Sample Preparation

The study enrolled women with singleton pregnancies at 5–9 weeks of gestation. Serum samples were collected in the morning after an overnight fast and stored at −80°C until use. In light of the evidence regarding the association between SCH and SA as mentioned earlier (7, 8), serum TSH, free T4 (FT4), thyroid peroxidase antibody (TPOAb), and thyroglobulin antibody (TgAb) were measured. Also, subclinical hypothyroid patients, based on TSH ≥ 2.5 mIU/L with a normal FT4 level and negative thyroid autoimmune antibodies, as well as normal control participants were selected for follow-up. A total of 82 individuals were finally divided into 4 groups: SCH patients (*N* = 24), SCH patients with SA (SCH + SA) (*N* = 21), SA patients (*N* = 19), and HCs (*N* = 18). Demographic characteristics, including age, gestation week (GW), history of SA, and body mass index (BMI), were also collected. In this study, we excluded multiple pregnancies; patients with a history of thyroid disease; and individuals with chronic systemic diseases, such as chronic renal failure, chronic heart failure, polycystic ovary syndrome, maternal reproductive anatomical abnormalities, other autoimmune diseases, or any medical drug interventions. The study was approved by the Ethics Committee of China Medical University (protocol no. ChiCTR-TRC-13003805). The study’s detailed protocol was explained, and written informed consent was obtained from every participant.

### Measurement of Thyroid Hormone and Thyroid Autoantibodies

Serum TSH, FT4, TPOAb, and TgAb were measured by an electrochemiluminescence immunoassay (Roche Diagnostics, Risch-Rotkreuz, Switzerland; Cobas Elecsys 601) (21). The minimum detectable concentration of TSH was 0.002 mIU/L. The intra-assay and inter-assay coefficients of variation were 1.57–4.12 and 1.26–5.76% (TSH), and 2.24–6.33 and 4.53–8.23% (FT4), respectively.

### Serum Processing and miRNA Prescreening

Before microarray analysis, we prepared serum pools using sera from patients in the 4 groups (4 from SCH + SA, 10 from SCH, 10 from SA, and 10 from HC groups) (22). Total RNA was extracted from a 200 µL serum pool using a mirVana miRNA isolation kit (Ambion/ThermoFisher Scientific, Naugatuck, CT, USA, #AM1556), according to the manufacturer’s instructions, followed by dephosphorylation, denaturation and labeling with Cyanine-3-CTP. After purification, the labeled RNAs were hybridized onto an Agilent Human miRNA microarray (8*60K, Design ID: 046064; Agilent Technologies, Santa Clara, CA, USA). After washing, the arrays were scanned with an Agilent Scanner G2505C (Agilent Technologies). Feature Extraction software (version 10.7.1.1, Agilent Technologies) was used to analyze array images and obtain raw data. Genespring software (version 12.5; Agilent Technologies) was employed to finish the basic analysis using the raw data. Differentially expressed miRNAs were then identified through any fold changes. The threshold set for up- and downregulated genes was a fold change ≥ 2.0. We randomly selected miRNAs differentially expressed only in one group with respect to other groups for further validation by real-time (RT)-polymerase chain reaction (PCR).

### Quantitative RT-PCR of Individual Serum miRNAs

Total serum miRNAs were isolated (Invitrogen, Carlsbad, CA, USA) as described previously (22, 23), with small modifications. Briefly, a volume of 10% SDS was added to the same volume of serum, vortexed, then incubated at room temperature for 1 h to better promote the separation of miRNAs from related protein factors. Proteins were then denatured in 3 volumes of Trizol solution to 1 volume of serum, vortexed, then incubated on ice for 10 min; by adding 1/5 volume of chloroform and sharply mixing for 15 s, aqueous and organic phases were separated after centrifugation at 12,000 rpm for 15 min at 4°C. The aqueous phase was carefully removed to a new tube; an equal volume of isopropyl alcohol was added and incubated at −20°C for 1 h. Total RNA precipitate was then separated after centrifugation at 12,000 rpm for 15 min at 4°C, followed by multiple washings in 75% precooled ethanol.

A MiRCURY LNA™ microRNA PCR System (Exiqon/Qiagen, Vedbaek, Denmark) was used to assess the presence of individual miRNAs in serum (24). Serum RNA-containing small RNAs were reverse-transcribed using a Universal cDNA Synthesis Kit II (Exiqon; #203301). Reverse-transcribed cDNA further served as a template for miRNA quantitative RT-PCR amplification reactions using an ExiLENT SYBR^®^ Green Master Mix Kit (Exiqon; #203421) in an ABI PRISM 7500 sequence detection system (Applied Biosystems, Waltham, MA, USA). Amplification conditions were: 10 min incubation at 95°C, followed by 40 cycles at 95°C for 15 s and 60°C for 1 min. The 10 µL reaction volume contained 5 µL of SYBR^®^-Green master mix, 1 µL of PCR primer mix and 4 µL of a diluted cDNA template. Syn-cel-miR-39 was added as a circulating miRNA normalizing control to monitor technical variations in RNA recovery and the effectiveness of RT-PCR and qPCR (25). All procedures were performed in accordance with the manufacturer’s protocol. Detailed information about primers for miRNAs is presented in Table 1. The relative expression of each miRNA was calculated using the comparative cycle threshold (CT) method (2^−ΔΔCT^) and Syn-cel-miR-39 as a normalized internal control (25).

**Table 1:** LNA™ polymerase chain reaction (PCR) primers used for qRT-PCR.

MicroRNA	Target sequence	Product no.
hsa-miR-197-5p (MIMAT0022691)	CGGGUAGAGAGGGCAGUGGGAGG	2102555
hsa-miR-33b-3p (MIMAT0004811)	CAGUGCCUCGGCAGUGCAGCCC	204462
hsa-miR-3960 (MIMAT0019337)	GGCGGCGGCGGAGGCGGGGG	2100264
hsa-miR-4443 (MIMAT0018961)	UUGGAGGCGUGGGUUUU	2104824
hsa-miR-4455 (MIMAT0018977)	AGGGUGUGUGUGUUUUU	2105370
hsa-miR-4515 (MIMAT0019052)	AGGACUGGACUCCCGGCAGCCC	2118009
hsa-miR-762 (MIMAT0010313)	GGGGCUGGGGCCGGGGCCGAGC	2114944
hsa-miR-940 (MIMAT0004983)	AAGGCAGGGCCCCCGCUCCCC	204094
hsa-miR-4530 (MIMAT0019069)	CCCAGCAGGACGGGAGCG	2105012
hsa-miR-486-5p (MIMAT0002177)	UCCUGUACUGAGCUGCCCCGAG	204001
hsa-miR-630 (MIMAT0003299)	AGUAUUCUGUACCAGGGAAGGU	204392
cel-miR-39 (MIMAT0000010)	UCACCGGGUGUAAAUCAGCUUG	203952

### Prediction of miRNA Target Genes and Functional Analysis

Using an miRWalk 1.0 online tool, target genes of differentially expressed miRNAs were further co-predicted with miRWalk, Targetscan, miRanda, PICTAR2, and DIANAmT software programs. The intersection sets of these five databases were used for bioinformatic analysis. Gene functions using gene ontology (GO) and Kyoto Encyclopedia of Genes and Genomes (KEGG) analyses were applied to determine the roles of such target genes using a DAVID Bioinformatics Resources 6.8 online tool for function annotation. The GO enrichment analysis included biological processes (BP), cell components, and molecular function (MF).

### Statistical Analysis

All statistical analyses were performed using SPSS 21.0 software (IBM Corp., Armonk, NY, USA). After normality analysis using a Shapiro–Wilk test, data that were or were not normally distributed were expressed as mean ± SEM, or median values and 25th–75th percentiles in brackets, and were compared by one-way repeated measures ANOVA or a Kruskal–Wallis test, respectively. Spearman’s correlations were performed to explore the relationships between miRNAs and TSH. Receiver-operating characteristic (ROC) curves analysis was performed to assess the ability of miRNAs to identify SCH + SA or SCH patients. The area under the ROC curve [area under the curve (AUC)] was calculated to evaluate the predictive power. All statistical tests were two-sided, and statistical significance was defined as *p* < 0.05. For GO and KEGG analysis, *p* < 0.05 was also considered to indicate a statistically significant difference (26).

## Results

### Screening Phase

**Figure 1:**
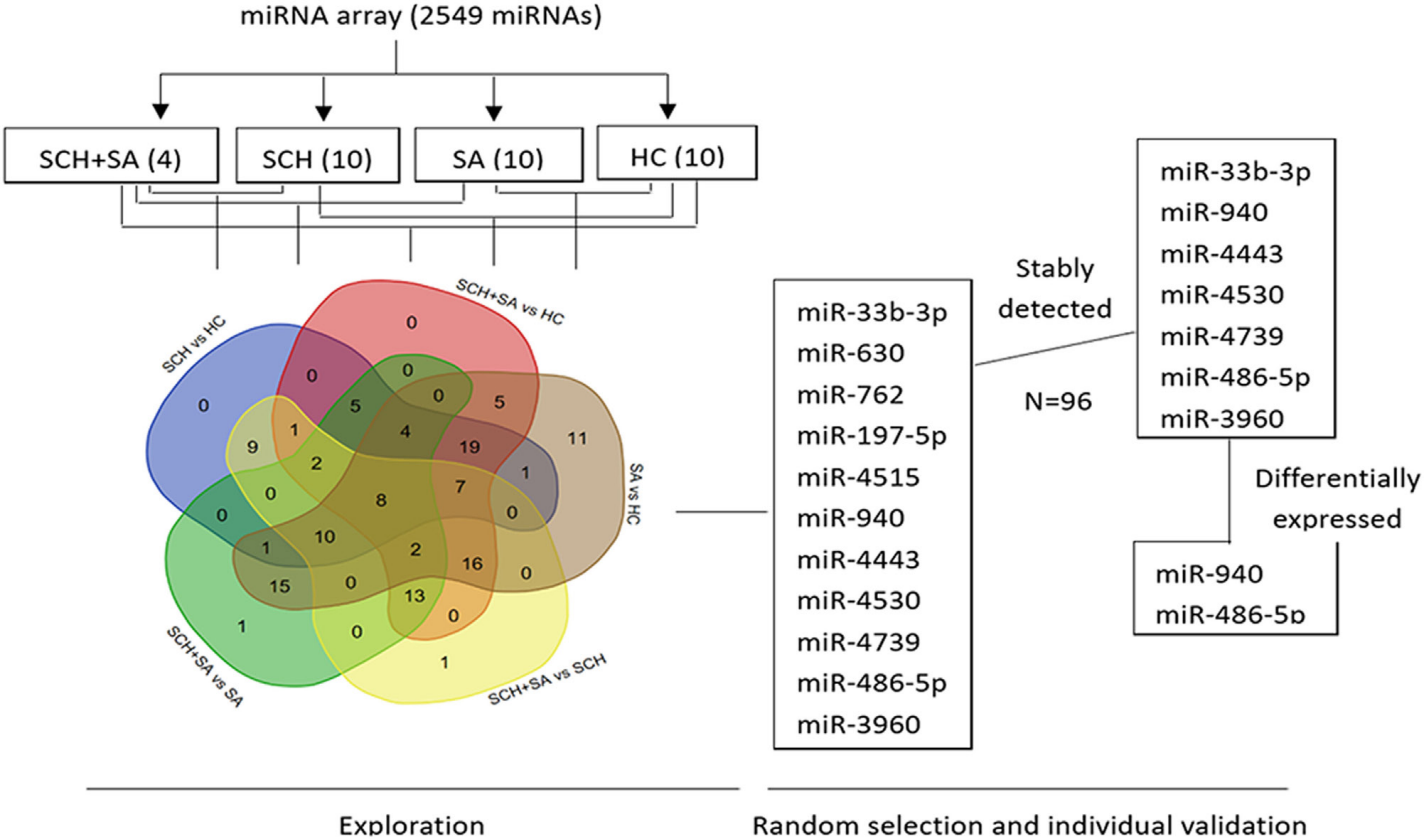
Flowchart of the screening process. Agilent Human microRNA (miRNA) microarray (8*60K, Design ID: 046064) representing 2,549 mature miRNAs was used to identify differentially expressed miRNAs among pooled sera [4 from the subclinical hypothyroidism (SCH) + spontaneous abortion (SA) group vs. 10 each, respectively, from SCH, SA, and healthy control (HC) groups]. A Venn diagram was drawn to better show the intersection of differentially expressed miRNAs between each set of two groups (131 miRNAs in total) using a web tool (http://bioinformatics.psb.ugent.be/webtools/Venn/). Of the 131 miRNAs that were differentially expressed (twofold alteration), 11 miRNAs were randomly selected for further individual validation. Seven of the 11 miRNAs were stably expressed among these 4 individual patients. Finally, two miRNAs were differentially expressed with a *p* < 0.05.

To identify miRNAs in serum from patients with SCH and/or SA, which may act as specific and reliable biomarkers for these diseases, pooled sera from 4 patients with SCH + SA, 10 patients with SCH, 10 patients with SA, and 10 patients with HC were analyzed for screening using an miRNA microarray. This pooling method for miRNA detection can reduce variation between individuals (24). As shown in Figure 1, of the 131 miRNAs that were differentially expressed (twofold alterations) after screening, 11 miRNAs were randomly selected for further external individual validation.

### Validation of miRNAs by RT-PCR and Clinical Characteristics of Patients

To validate potential biomarkers identified from screening results, serum levels of miRNAs were measured by qRT-PCR in 24 patients with SCH, 21 patients with SCH + SA, 19 patients with SA, and 18 patients with HC. Clinical parameters and patient demographics are displayed in Table 2. Except for TSH levels, significant differences were not found in clinical factors, including maternal age, gestational week, BMI, abortion history, and FT4 levels.

**Table 2:** Characteristics of patients.

	SCH + SA	SCH	SA	HC	*p* Value
Maternal age (years)^a^	29 (27–31)	30 (26.75–31)	27 (26–30)	29 (26.25–34)	0.58^b^
GW (weeks)^c^	6.34 ± 0.19	6.55 ± 0.16	6.73 ± 0.27	6.98 ± 0.20	0.182^d^
BMI (kg/m^2^)^c^	21.47 ± 0.55	22.38 ± 0.63	22.49 ± 0.65	21.54 ± 0.69	0.542^d^
AH (*n*/*N*)	(5/25)	(3/26)	(9/23)	(9/22)	0.054^e^
FT4 (pmol/L)^c^	16.05 ± 0.29	16.19 ± 0.34	17.02 ± 0.30	16.4 ± 0.34	NA
TSH (mIU/L)^a^	3.80 (3.39–5.04)	3.94 (3.27–5.26)	1.44 (1.01–2.08)	1.57 (1.23–1.94)	NA

*GW, gestational week; BMI, body mass index; AH, abortion history, including spontaneous abortion and medical abortion history; NA, not applicable; FT4, free T4; TSH, thyroid-stimulating hormone; SCH, subclinical hypothyroidism; SA, spontaneous abortion; HC, healthy control*.

*^a^Values are expressed as mean ± SEM*.

*^b^Kruskal–Wallis test*.

*^c^Values are expressed as median values and 25th–75th percentiles in brackets*.

*^d^One-way ANOVA test*.

*^e^Chi-square test*.

**Figure 2:**
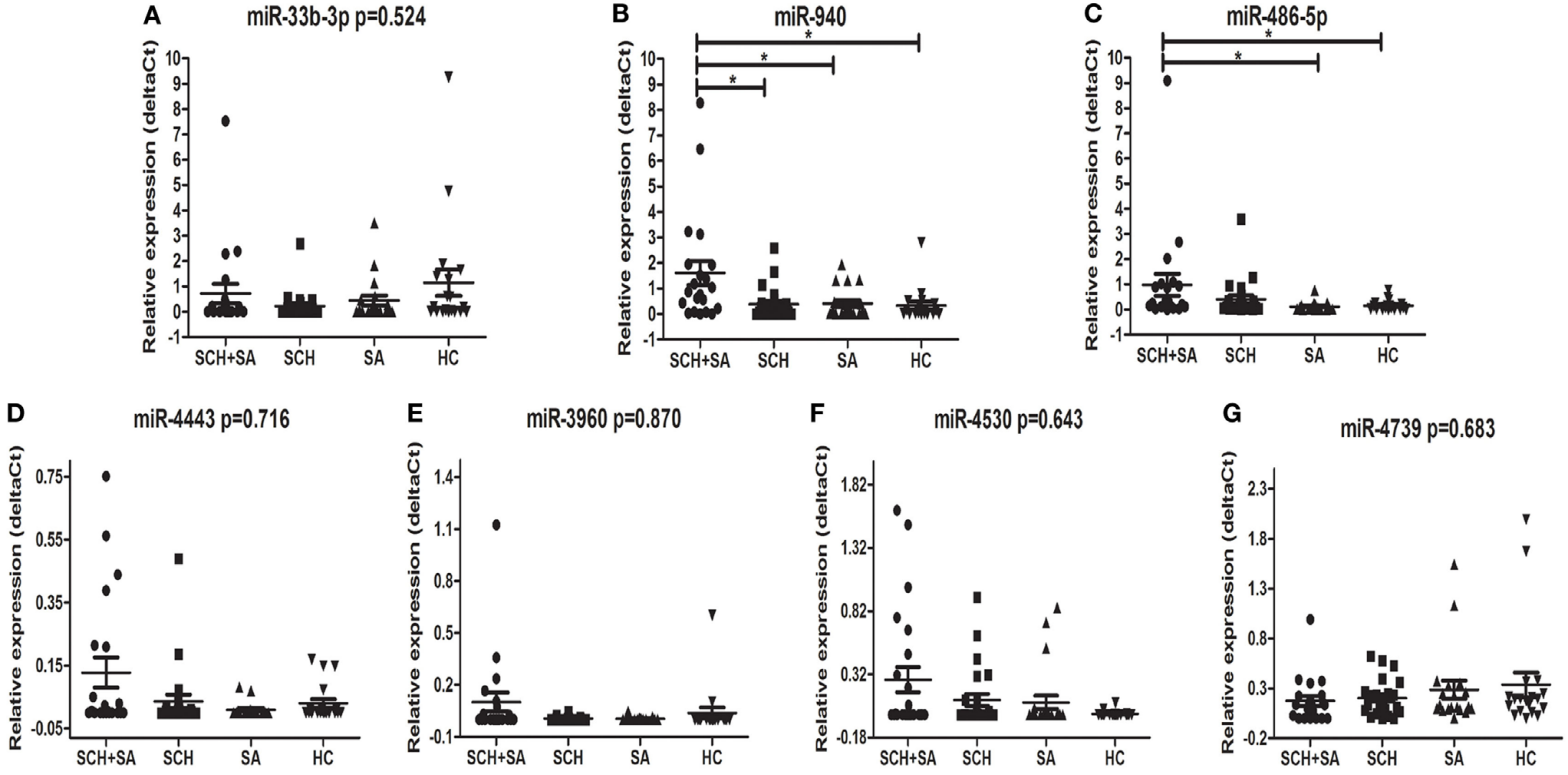
Comparison of the serum levels of miR-33b-3p **(A)**, miR-940 **(B)**, miR-486-5p **(C)**, miR-4443 **(D)**, miR-3960 **(E)**, miR-4530 **(F)**, and miR-4739 **(G)** in subclinical hypothyroidism (SCH) + spontaneous abortion (SA), SCH, SA, and healthy control (HC) groups. Syn-cel-miR-39 served as the spike-in control to monitor the effectiveness of real-time (RT)-polymerase chain reaction (PCR) and quantitative (q)PCR. The serum levels were determined using RT-PCR following RNA extraction. A Kruskal–Wallis test was performed to determine the significance of differences between groups; *p < 0.05 was considered significant.

Only 7 of 11 miRNAs, including miR-33b-3p, miR-940, miR-4443, miR-4530, miR-4739, miR-486-5p, and miR-3960, were stably detected in all 4 groups (Figure 1). In the qPCR analysis, the relative expression of miR-940 showed a significant upregulation in patients with SCH + SA compared with those with SCH or SA, and HC (*p* < 0.05, Figure 2). Significant differences in miR-940 expression were not found between patients with SCH and HC or between patients with SA and HC. As displayed in Figure 2, miR-486-5p significantly increased in patients with SCH + SA compared with patients with SA or HC (*p* < 0.05 for both), but was only slightly elevated compared with patients with SCH (*p* > 0.05). The miR-486-5p expression also showed no significant differences either between HC and patients with SCH or between SA and HC groups. We did not find any significant differences in the other five miRNAs, which may due to the small sample sizes used in the screening phase.

### Biological Functions of Differentially Expressed Candidate miRNAs as Predicted Targets

**Figure 3:**
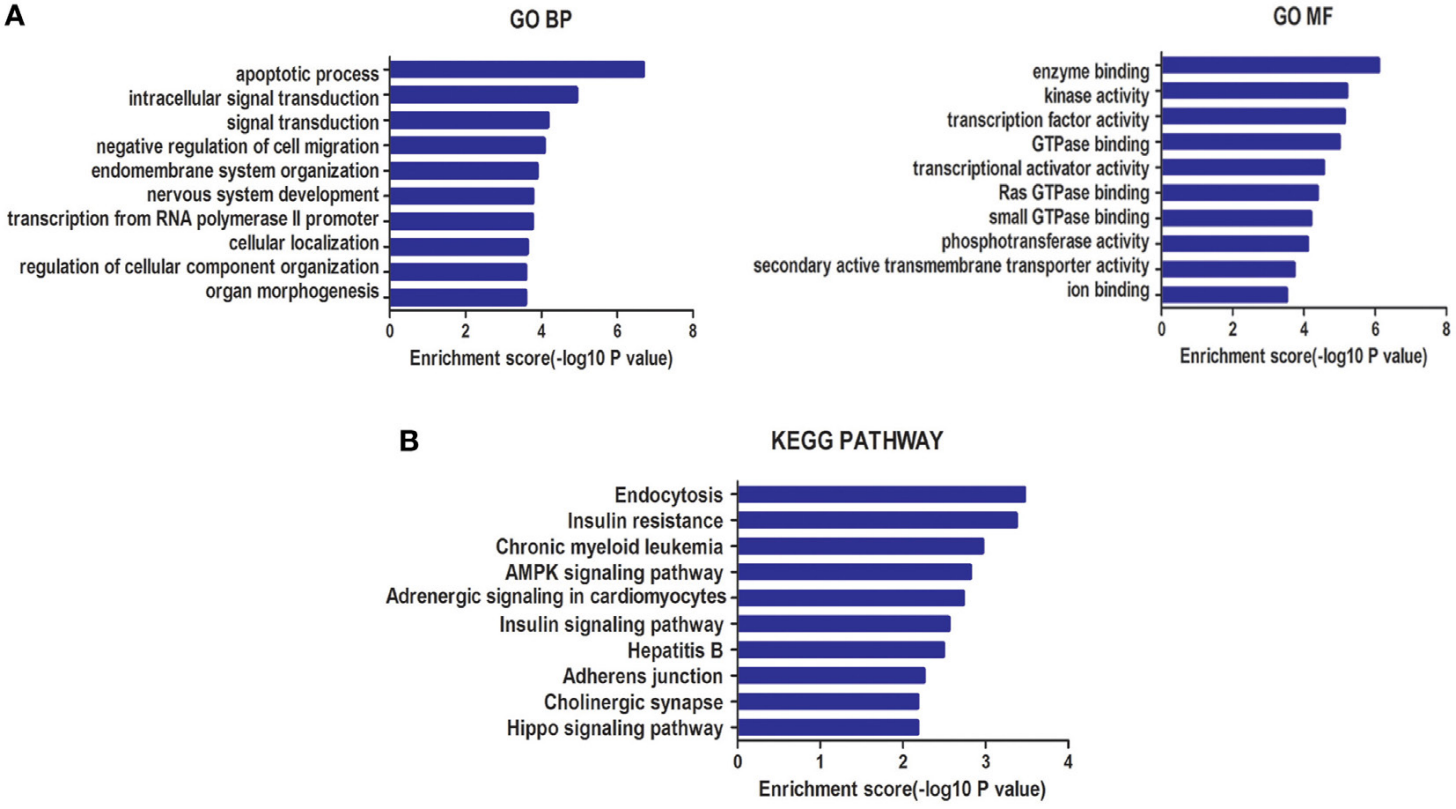
**(A)** Gene ontology (GO) analysis for genes targeted by two types of differentially expressed microRNAs (miRNAs): biological processes (BP) and molecular function (MF). **(B)** Kyoto Encyclopedia of Genes and Genomes (KEGG) pathway enrichment analysis for genes targeted by these two types of miRNAs. Both enrichment scores were calculated as the negative logarithm of a *p* value (−Log *p*). The top 10 significantly enriched items are listed for each level.

A total of 2,655 target genes of miR-940 and 995 target genes of miR-486-5p, co-predicted with the five software programs used, were selected for GO enrichment and KEGG pathway analyses. GO analysis indicated that the target genes of these miRNAs were significantly enriched in BP, such as the apoptotic process and the negative regulation of cell migration, and in MF, such as enzyme binding and kinase activity (Figure 3A). In the KEGG pathway analysis, an enrichment of genes occurred in pathways such as insulin resistance, AMPK signaling, and Hippo signaling (Figure 3B).

### Correlations Between Candidate miRNAs and TSH

**Figure 4:**
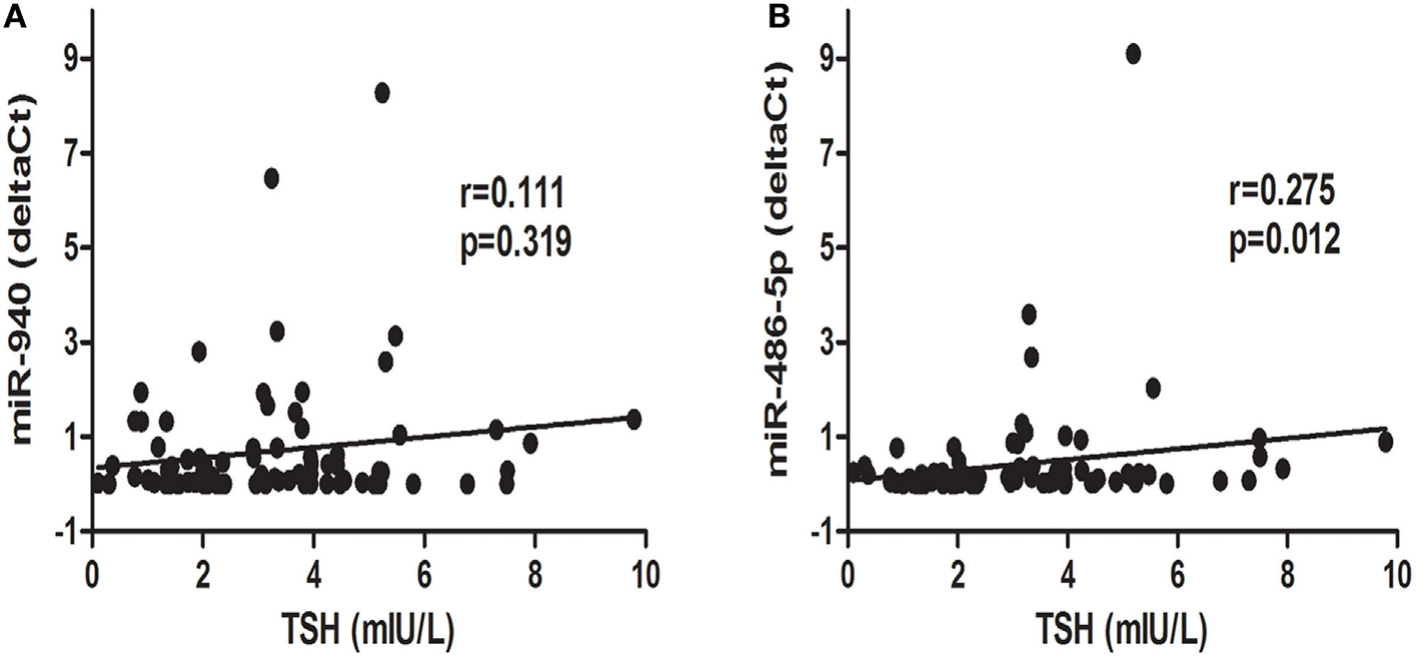
Correlation analysis of differentially expressed microRNAs, miR-940 **(A)** and miR486-5p **(B)**, and thyroid-stimulating hormone (TSH) levels; *p* < 0.05 was considered significant.

To further explore the relationship between candidate miRNAs and TSH levels, a correlation analysis was conducted. Interestingly, as displayed in Figure 4, miR-486-5p expression was positively correlated with TSH levels (*r* = 0.275, *p* < 0.05), whereas an association between serum miR-940 and TSH levels was not found (*p* > 0.05).

### The Ability of Each miRNA to Distinguish SCH + SA From Other Groups or All SCH Subjects From All Non-SCH Groups

**Figure 5:**
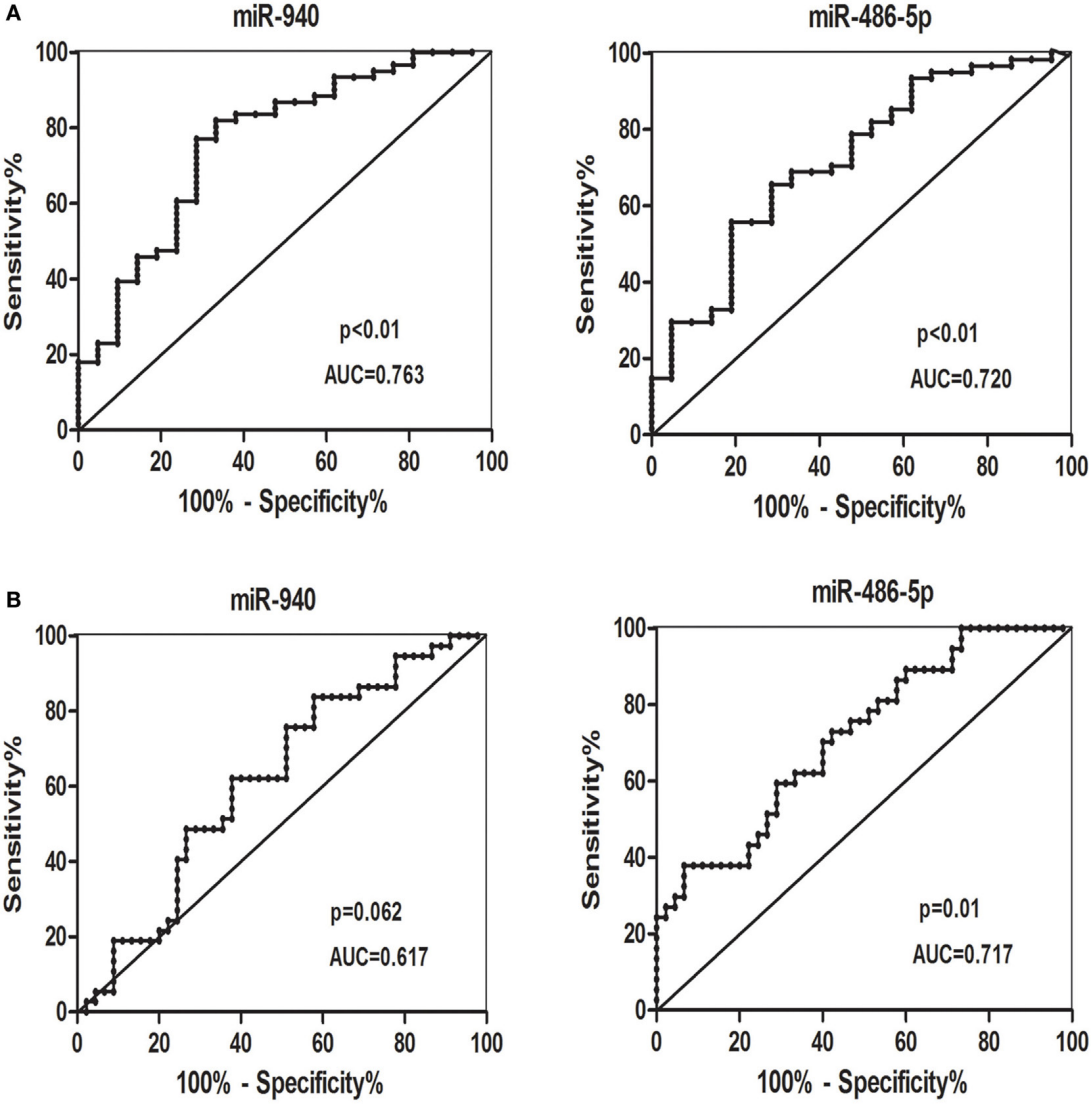
**(A)** Receiver-operating characteristic (ROC) curves for the ability of the differentially candidate serum microRNAs (miRNAs) to differentiate the patients with subclinical hypothyroidism (SCH) + spontaneous abortion (SA) from the other groups [SCH, SA, and healthy control (HC)]. **(B)** ROC curves for the ability of differentially expressed candidate serum miRNAs to differentiate all patients with SCH (SCH + SA and SCH) from the other groups (SA and HC). Abbreviation: AUC, area under the curve.

Receiver-operating characteristic curves were conducted to estimate the sensitivity and specificity of candidate serum miRNAs to distinguish patients with SCH + SA from other groups, or all patients with SCH from all non-SCH groups. miR-940 and miR-486-5p (Figure 5A) had the following areas under the ROC curve: 0.763 (*p* < 0.01) and 0.720 (*p* < 0.01), respectively, which distinguished patients with SCH + SA from the other groups (SCH, SA, and HC). As Figure 5B demonstrates, miR-486-5p had an AUC (0.717, *p* = 0.01), which distinguished all patients with SCH (SCH + SA and SCH) from the other groups (SA and HC); however, miR-940, with an AUC of 0.617 (*p* > 0.05), could not be used make this distinction.

## Discussion

Evidence has shown that miRNAs in serum may act as potential molecular biomarkers in abortion (20) and may also be influenced by TSH (25). This is the first study focused on characterizing serum miRNA profiles in patients with SCH and SA after excluding the interference of autoantibodies. In our study, we screened and analyzed miRNAs in patients with SCH, SCH + SA, or SA and HC. We showed that miR-940 was significantly more elevated in patients with SCH + SA than in patients with SCH or SA, or HC. In addition, the level of miR-486-5p was significantly lower in patients with SCH + SA than in patients with SA or HC, but was only slightly more elevated than in patients with SCH. The function of predicted target genes of these two differentially expressed miRNAs were evaluated by GO and KEGG pathway analyses. In addition, using miR-940 and miR-486-5p alone, we could distinguish patients with SCH + SA from the SCH, SA and HC groups. miR-486-5p can also be used to distinguish all patients with SCH (SCH + SA and SCH) from the other two groups (SA and HC). These results indicate the potential diagnostic value of serum miRNAs for SCH and abortion.

In this study, we identified an elevated level of miR-486-5p in cases of SCH but not SA. The expression of miR-940 was also significantly elevated in patients with SCH + SA compared with those with SCH or SA, and HC. miR-486 has been widely researched, being found to be significantly overexpressed in prostate cancer, and may play a causative role in prostate cancer by promoting cellular proliferation, migration, and colonization (27). Other studies have shown that miR-486 can mediate tumor-suppressive effects by targeting fibrillin 1 in papillary thyroid carcinoma (28), by targeting CDK4/BCAS2 protein in esophageal cancer (29), and by targeting the insulin-like growth factor 1 receptor and its downstream effectors, the mammalian target of rapamycin, the signal transducer, and activator of transcription 3 and c-Myc, in hepatocellular carcinoma (30). miR-486 has also been linked with metabolism. miR-486 may accelerate preadipocyte proliferation and myotube glucose intolerance, and circulating miR-486 in serum can be used for predicting the future risk of adult type 2 diabetes in obese children (31). miR-486, prevailing in high-density lipoprotein 2, can be used as an additional tool to identify vulnerable coronary artery disease patients (32). miR-486 may also be pregnancy-related. Expression levels of miR-486-5p in whole blood samples increased during pregnancy in pregnant mares (33). The expression of miR-486-3p in placental villous is significantly lower in women with recurrent miscarriage than in gestational age-matched normal pregnancies. The level of miR-486-3p was significantly higher at implantation than at non-implantation sites in mice, indicating its involvement in embryo implantation (34).

As for miR-940, its upregulation by transfection with miR-940 vector can inhibit cell proliferation and induce apoptosis by targeting PKC-δ in ovarian cancer (35). miR-940 may suppress tumor cell invasion and migration *via* regulation of the migration and invasion enhancer 1 protein in prostate cancer (36), the inhibition of phosphorylation of myosin II in breast cancer (37), and the regulation of CXC chemokine receptor 2 in hepatocellular carcinoma (38). However, overexpression of miR-940 promoted cell migration, invasion, and metastasis by repression of zinc finger protein 24 expression in gastric cancer (39), and accelerated cell growth, proliferation and cell cycle arrest by the inhibition of p27, phosphatase and tensin homolog in cervical cancer (40). There is no evidence concerning the role of miR-940 in miscarriage or SCH as yet. However, a limitation exists regarding the number of participants involved, which may result in a consistently wide variation. In addition, diet may also play a role in changes in circulating miRNA (41–43), thyroid autoimmunity in pregnancy, and postpartum (44) and adverse pregnancy outcomes (45), which may inevitably influence our findings. These should be items to focus on in the study of SCH-related abortions in future.

The GO analysis showed that the target genes of these miRNAs were intensively involved in the apoptotic process and negative regulation of cell migration during BP, and in enzyme binding and kinase activity during MF that may contribute to embryo implantation, uterine decidualization and placentation for the maintenance of pregnancy. For example, trophoblast cells share the same invasive characteristic with carcinoma cells (46). In KEGG pathway analysis, genes involved in insulin resistance, and AMPK and Hippo signaling pathways were enriched. SCH has been linked with insulin resistance, even at a high–normal level (47). AMPK signaling, one of the stress-related pathways, plays a pivotal role during implantation by combating stress responses such as hyperinsulinemia (48). Components of the Hippo signaling pathway, such as Yes-associated protein 1, which plays a crucial role in cell proliferation, differentiation, apoptosis, and development, were reported to mediate human decidualization of uterine endometrial stromal cells (49).

We analyzed the expression of significantly dysregulated miRNAs as potential biomarkers of SCH or SA. ROC curves revealed that the two miRNAs alone show a promising ability to distinguish patients with SCH + SA from the SCH, SA and HC groups. miR-486-5p can also be used to distinguish all patients with SCH (SCH + SA and SCH) from the other two groups (SA and HC). This is the first time that serum miRNAs have been shown to act as useful predictive biomarkers in SCH-related SA. Moreover, only miR-486-5p showed significant correlation with TSH levels, suggesting that further validation of the relationship between miR-486-5p and TSH is needed. Correlations between miRNAs and baseline characteristics, including age, GW, and BMI, were not found which is consistent with the findings of previous studies (25).

In conclusion, our study first identified two miRNAs (miR-486-5p and miR-940) that showed differences in serum levels in SCH, SA, and SCH combined with SA groups of patients. Bioinformatic and ROC analyses suggest a role for miRNAs in the pathogenesis of SA in women with SCH and the potential utility of miRNAs in predicting SCH and SA. However, more studies are warranted to determine the potential value of circulating miRNAs of SA in patients with SCH.

## Ethics Statement

The study was approved by the Ethics Committee of China Medical University. Informed consent was obtained from participants in advance.

## Author Contributions

YiZ and ZS: Conception and design. YiZ, XW, YuZ: Acquisition, analysis, or interpretation of data. YiZ: Performance of experiments and drafting of manuscript. ZS: Critical revision. ZS and WT: Final approval of the version to be published.

## Conflict of Interest Statement

The authors declare that the research was conducted in the absence of any commercial or financial relationships that could be construed as a potential conflict of interest.
